# Rasch analysis of the Psychiatric Out-Patient Experiences Questionnaire (POPEQ)

**DOI:** 10.1186/1472-6963-10-282

**Published:** 2010-09-28

**Authors:** Rolf V Olsen, Andrew M Garratt, Hilde H Iversen, Oyvind A Bjertnaes

**Affiliations:** 1 Norwegian Knowledge Centre for the Health Services, Boks 7004, St. Olavs plass, 0130 Oslo, Norway; 2 Department of Teacher Education and School Research, University of Oslo, Norway

## Abstract

**Background:**

The Psychiatric Out-Patient Experiences Questionnaire (POPEQ) is an 11-item core measure of psychiatric out-patients experiences of the perceived outcome of the treatment, the quality of interaction with the clinician, and the quality of information provision. The POPEQ was found to have evidence for reliability and validity following the application of classical test theory but has not previously been assessed by Rasch analysis.

**Methods:**

Two national postal surveys of psychiatric outpatients took place in Norway in 2004 and 2007. The performance of the POPEQ, including item functioning and differential item functioning, was assessed by Rasch analysis. Principal component analysis of item residuals was used to assess the presence of subdimensions.

**Results:**

6,677 (43.3%) and 11,085 (35.2%) psychiatric out patients responded to the questionnaire in 2004 and 2007, respectively. All items in the scale were retained after the Rasch analysis. The resulting scale had reasonably good fit to the Rasch model. The items performed the same for the two survey years and there was no differential item functioning relating to patient characteristics. Principal component analysis of the residuals confirmed that the measure to a high degree is unidimensional. However, the data also reflects three potential subscales, each relating to one of the three included aspects of health care.

**Conclusions:**

The POPEQ had excellent psychometric properties and Rasch analysis further supported the construct validity of the scale by also identifying the three subdimensions originally included as components in the instrument development. The 11-item instrument is recommended in future research on psychiatric out-patient experiences. Future development may lead to the construction of more precise measures of the three subdomains that the POPEQ is based on.

## Background

In recent years there has been a steady growth in the availability of quality indicators that are designed to inform patients, providers and policy makers about the quality of health care provision [1,2]. In addition to traditional clinical measures of outcome, questionnaires are increasingly used to assess the perceptions of health professionals and patients in relation to health care quality [3].

The measurement of patient experiences and satisfaction is recognized as an important part of health care evaluation, quality indicators and performance measurement. Patient experiences and satisfaction are central to the WHO's framework for assessing the performance of health systems [4], it is included as one of three core quality dimensions in the OECD quality indicator framework [5], and a large number of national and cross-national surveys of patient experiences have been conducted [6]. The results of surveys can contribute to quality improvement, public accountability and transparency [7]. However, several methodological challenges threaten the value of using data from patient experience surveys that include the psychometric properties of measurement instruments [8], non-response [9], and case-mix [10,11].

The measurement of patient experiences or satisfaction is based on self-report where individual patients respond to a scale to reflect their perceptions of health care quality. Patient responses are considered as indicative of one or several unobserved latent traits. The general principles for the measurement of psychological constructs often referred to as psychometrics or test theory, provide the underlying methods for constructing a scale reflecting such latent traits. However, a review of 195 patient satisfaction articles found that the satisfaction instruments had little evidence of reliability or validity, casting doubt on the credibility of findings [8]. The review included articles describing questionnaire development and testing, but such studies usually apply traditional procedures derived from classical test theory (CTT). Alternative approaches to deriving measures of psychological attributes that have been formalized mathematically have been available for several decades. At the most general level such models may be labeled as latent trait models including for instance structural equation models and more relevant for this paper, item-based approaches such as item-response theory (IRT) and Rasch models. The great advantage of these models is the fact that they are explicit empirical models of the latent trait allowing for testing of fit between the data and a theoretical model. The vast majority of health-related research that has used IRT relate to the development and evaluation of measures for health status and quality of life, and only a handful of articles have reported the use of IRT approaches in the analysis of scales developed to measure patient experiences or satisfaction [12-17].

This article applies the Rasch model [18], as a supplement to CTT in order to assess the psychometric properties of one of the measures derived from the Psychiatric Out-Patient Experiences Questionnaire (POPEQ). The questionnaire has been used in two consecutive national patient experience surveys in Norway [19,20]. The development of the questionnaire followed a literature review including widely used questionnaires within psychiatry that was designed to identify domains and items of potential relevance to psychiatric outpatients [21-27]. The review showed that Norway lacked a standardized, validated questionnaire for the measurement of outpatients' experiences with mental health care in Norway. Therefore development work was undertaken which included reviews of items by an expert group, cognitive interviews with patients and piloting [19]. Following qualitative interviews with patients and consultation with an expert group, the items and domains identified by the review were assessed for relevance and supplemented by additional items and domains. This process was designed to ensure the content validity of the POPEQ. The core 11-item POPEQ includes a range of patient experiences questions relating to the three domains of perceived outcome of the treatment (3 items), the quality of interaction with the clinician (5 items), and the quality of information provision (3 items) in addition to a number of single items and background questions. The three domains are often included in other psychiatric patient experiences questionnaires, and most domains covered in a validated Swedish outpatient questionnaire for psychiatric patients were also part of the POPEQ [25].

The validity and reliability of the original measure based on classical test theory was assessed and reported following a national survey in 2004 [19] The work that follows uses the same analyses for the 2007 data and compares the results with those for 2004. The pooled datasets are then tested using Rasch analysis which includes assessing how the items function according to the general principles of Rasch analysis and how well each of the item response categories differentiate between patients estimated to be at different levels of the latent variable. Further, by assessing differential item functioning (DIF) within the Rasch model, the invariance of the items in relation to several respondent characteristics is assessed.

In addition, a more explicit test for the assumption of unidimensionality is undertaken. The original 11-item measure is broad in nature reflecting three different aspects of the quality of the services, and as such, the measure may include several sub-dimensions. A translated version of the 11 items may be accessed as additional file 1 to this manuscript.

## Methods

### Data collection

The POPEQ was included in a self-completed questionnaire that was mailed to the homes of patients aged 18 years and over. In the 2004 survey, the POPEQ was mailed to 15,422 patients from 90 clinics across Norway. Patients who had an outpatient visit in September 2004 were mailed a questionnaire by mid October and non-respondents were mailed a reminder questionnaire after three weeks. In the 2007 survey, the POPEQ was mailed to 31,482 patients from 100 clinics. Patients were included in two strata, patients visiting the clinic from the 20^th ^of August - 30^th ^of September and the 1^th ^of October - 10^th ^of November, respectively. The patients were sent a questionnaire during the first half of the next month. Reminders were sent to non-respondent three weeks later and after six weeks.

The study was approved by the Norwegian Regional Committee for Medical Research Ethics, the Data Inspectorate and the Norwegian Board of Health.

### Statistical analysis

**Figure 1:**
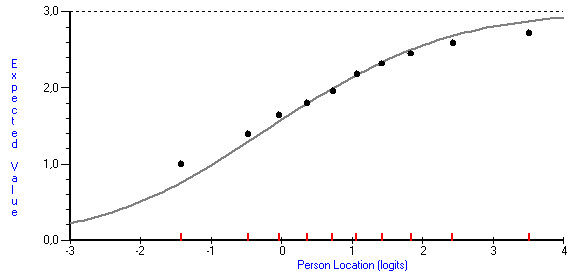
**Example of an item characteristic curve for one item**. Item characteristic curve for the item: "Perceived outcome of the treatment: Change in psychological problem".

Rasch analysis is an item-based approach where ordinal observed item scores are transformed to linear measures representing the underlying latent trait [28]. Rasch analysis is based on a mathematical model where the probability for endorsing an item at different levels of the response scale is a logistic function of the difference between the person and item location on the scale [29,30]. In graphical form, these logistic functions are referred to as item characteristic curves. Figure 1 gives one example of the curve for one of the items in the instrument.

At the core of the Rasch model is the requirement of *invariant *measurement. In brief, invariance refers to the fact that the scores for persons should not be a function of specific items, and vice versa, item parameters should not be affected by specific persons in the sample [18]. In the Rasch model item and person parameters are located on the same scale. The unit of this scale is usually referred to as the *logit*. The origin of the scale is usually set to the average location for the items included.

In addition to locating the person and item parameters on the same linear scale Rasch analysis has several advantages as compared to classical test theory: items are compared against a formal mathematical model; scores for the respondents can be computed without replacing missing values; and, the requirement that a measure should only capture one single dimension can be tested more explicitly [28].

There are several versions of the Rasch model, and the model used in this study is the partial credit model [31] which is applicable to item sets that are polytomously scored [32]. Mathematically, the model is expressed as:

$$P\{X_{ni}=x\}=\frac{e^{(x(\beta_{n}-\delta_{i})-\sum_{k=1}^{m}\tau_{ki})}}{\sum_{x=0}^{m}e^{(x(\beta_{n}-\delta_{i})-\sum_{k=1}^{m-1}\tau_{ki})}}$$

*P*{*X*_*xi *_= *x*} is the probability for person *n *with the person parameter *β_n _*receiving *x *points on item *i *with parameter *δ_i_*, where *xε*{0,1,2, ..., *m*}. In this context the parameters *β_n _*are measures of the persons' level of appraisal of the services offered, and *δ_i _*express how hard it is to endorse the statement in item *i*.

An item with *m + 1 *ordered response categories has *m *thresholds *τ_k _*where *kε*{1,2,...*m*}. It is possible to interpret the thresholds *τ_k _*as "parameters *δ_ik _*for each response category" for item *i*. The thresholds that define the score categories are the points on the latent scale where the (conditional) probabilities of scoring in one of two adjacent categories are equal: The threshold value *τ_1 _*corresponds to the level of appraisal with which it is 50% likely to score 1 point rather than 0. The threshold value *τ_2 _*corresponds to the level of appraisal with which it is 50% likely to score 2 points rather than 1 point.

Five response categories (0, 1, 2, 3 or 4 points) result in four thresholds which should be ordered, that is *τ*_*ki *+ 1 _>*τ*_*ki*_. If items have unordered thresholds, they should be rescored by collapsing adjacent categories [33].

In this article we use Rasch analysis to corroborate the evidence that POPEQ functions as a valid and reliable measure of psychiatric out-patients experiences with their clinics [19]. The degree of fit of the POPEQ to the Rasch model was assessed using the software Winsteps [34] and the outfit and infit mean square residuals. These fit indexes are both based on mean squares of the deviation between the data and the model, the only difference being that the infit statistics is a weighted estimate where persons close to the item location is given more weight than those at the tails of the distribution. Both statistics have an expected value of 1 if the data fits the model. Values above 1 indicate underfit or low discrimination, and values below 1 indicate overfit or high discrimination. As a rule of thumb Linacre [35] suggests that mean squares in the interval 0.5 - 1.5 may be regarded as *productive for measurement*. In this paper we do not report t-statistics for these mean squares since the sample size is very large and hence any deviation between model and data would be reported as a statistically significant deviation [36].

If items live up to the requirement of invariant measurement patients with the same person location will have the same probability of endorsing an item, independent of the subgroups to which they belong. One particular case of violation of this requirement is differential item functioning (DIF), a situation where items functions differently for patients in different subgroups, e.g. male and female. Patients with the same value on the latent trait should have the same probability for endorsing an item, independent of persons characteristics such as gender, age etc. DIF was assessed using the ANOVA test within RUMM 2020 [37-39]. The samples in this study are very large and hence the smallest deviations between data and the model are statistically significant. Therefore, the DIF analysis is based on adjusting the chi squares to a sample size of 500. This is helpful in order to identify deviations between model and data of practical significance [36]. DIF was assessed in relation to the most important predictors of patient experiences in national reporting; age under or over 40 years, gender, diagnosis dichotomised according to severity, under or over 4 consultations and survey year [20]. The latter DIF-test, the one for survey-year, is of particular relevance since the POPEQ surveys are repeated at regular intervals. By conducting DIF analysis across survey years it is possible to assess whether items are drifting, that is, to test if items relate consistently to the construct across time.

For the Rasch model to be applicable, the items must contribute to a unidimensional scale for which the POPEQ has evidence based on the results of factor analysis [19]. However, factor analysis of the raw responses may be problematic since the raw responses are non-linear, ordinal data [40]. Following previous evaluations of patient-reported outcome measures using Rasch analysis [41], principal component analysis (PCA) of the person-by-item residuals was undertaken to assess the dimensionality of the measure. The dimensionality was assessed by inspecting the eigenvalues and factor loadings of the PCA components. In addition, as proposed by Smith [42], scores were calculated for any subdimensions suggested by PCA and independent t-tests of the equality of the mean scores conducted to assess the effect of potential multidimensional structure in the data. As a rule of thumb Tennant & Conaghan [28] have suggested that if more than 5% of the persons have significantly different subdimension scores, this is indicative of the presence of two or more subdimensions in the data.

## Results

### Data collection

All patients who had responded to at least one of the eleven items were included in the analysis. In 2004 respondents differed from non-respondents in relation to age and gender. These differences were statistically significant [19]. In 2007, the respondents differed from non-respondents in relation to age, gender, diagnosis and the number of consultations in the inclusion period. To assess nonresponse bias 293 postal non-respondents from 10 clinics were randomly selected to be included in a telephone follow-up. 110 patients answered by telephone and the difference between telephone respondents and postal respondents on the POPEQ-11 scale was small and insignificant (2 on a scale from 0 to 100), indicating little non-response bias [20].

Table 1 show that the 2007 response rate was lower than in 2004, and the proportion of respondents with only one consultation was almost doubled from 2004 to 2007. There were no significant differences in the amount of missing data between the two surveys. In total there was 4.4% missing data with most relating to three items where it was possible to respond "Not applicable".

**Table 1:** Sample characteristics for the 2004 and 2007 surveys

	**2004**	**2007**
		
N (response rate)	6 677 (43.3%)	11 085 (35.2%)
Proportion male	32.1%	30.8%
Proportion higher ed.	29.0%	35.5%
Mean age (st. dev.) in years	39.5 (12.4)	41.0 (12.7)
Proportion one consultation	7.7%	14.5%

### Statistical analysis

When replicating the analyses based on classical test theory for the 11 items, results were very similar for the 2004 and 2007 surveys. The results of factor analysis and tests for internal consistency shown in Table 2 further confirm that the POPEQ scale raw scores are internally consistent and essentially unidimensional. Cronbach alpha is above 0.9 and one dominant first factor accounts for more than 50% of the variance. The items were in general marginally less endorsed in 2007 (data not shown).

**Table 2:** Main outcomes of the classical test theoretical analysis.

	**2004**	**2007**
		
Mean score (st.dev.) 0-100	68.7 (18.3)	67.3 (19.7)
Cronbach's alpha	0.91	0.92
Test-retest reliability	0.90	
Variance 1^st ^factor	53.7%	57.3%

**Figure 2:**
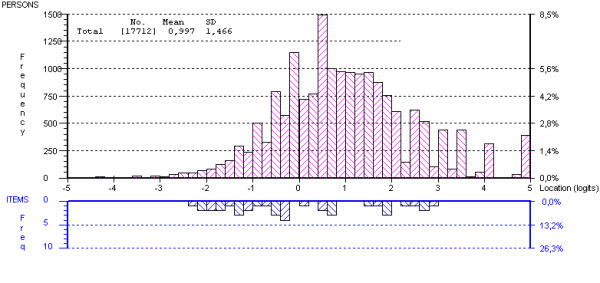
**Item thresholds by person locations**. Distribution of item thresholds (lower half) and person locations (upper half) along the scale. Grouping is set to interval length of 0.20.

Table 3 shows the summary statistics for the Rasch analysis. The person locations are skewed somewhat towards positive experiences with care. This is also reflected in the person-item threshold distribution shown in Figure 2. This is regularly seen for measures of patient experience or satisfaction; most patients report positive experiences with their health care. Nevertheless, the scale is very successful in separating persons at different levels of the scale as indicated by the person separation index.

**Table 3:** Summary statistics for the Rasch analysis

Mean (SD) item location	0	(1.62)^1^
Mean (SD) person location	1.04	(1.54)
Person separation index	0.91	

^1 ^SD represented by the standard deviation of the threshold locations which better reflects the spread of item difficulties.

Table 4 shows the item parameters and the outfit and infit mean squares. Patients at the lower end of the scale report that their psychological problems were reduced after the treatment, and they also report that they are given time to talk with their clinician. At the other end of the scale, the item locations reveal that high levels of appraisal are particularly related to positive evaluations of the quality of the information provided.

**Table 4:** Item analysis

Item no	**Content of the questions**^**1**^	Location	Outfit mean square	Infit mean square
	*Perceived outcome of the treatment*			
11	Conversation with professional	-0.16	0.9	0.9
12	Overall treatment outcome	0.09	0.8	0.8
13	Change in psychological problem	-0.46	1.5	1.3
				
	*Quality of interaction with the clinician*			
18	Enough time for contact/dialogue	0.07	1.0	1.0
19	Understanding	-0.44	0.7	0.8
20	Therapy/treatment suitability	0.04	0.7	0.7
21	Follow-up actions carried out	-0.26	0.9	0.9
22	Communication	-0.56	1.1	1.2
				
	*Quality of information provision*			
23	Patient say in treatment package	0.60	1.2	1.2
24	Treatment options	0.62	1.2	1.1
25	Psychological problems	0.46	1.1	1.1

^1 ^See additional file 1 for a complete translation of the questions and the response options

Two items had disordered thresholds which was resolved by collapsing the two lowest categories which did not discriminate sufficiently [33]. The mean squares are within the interval 0.5 - 1.5 which implies that the items fit reasonably well to the model and they may be regarded as useful for measurement [35]. The item relating to the patient's perception that the psychological problem is reduced after the treatment had an outfit mean square slightly outside this range. The item characteristic curve (ICC) for this item is given in Figure 1. The figure shows that the item discriminates marginally less than required by the Rasch model. However, as can be seen by the figure, this problem is mainly due to lack of discrimination at the two tails of the distribution, while for respondents who are around the level targeted by the item, the item performs reasonably well. This is also reflected in the relatively lower infit mean square.

**Figure 3:**
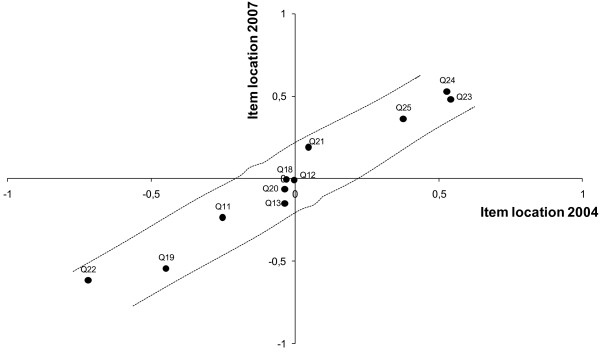
**Scatterplot of item locations in 2004 and 2007**. Common item linking 2004 and 2007 with the paired 95% quality control lines. Items within the two quality control lines functions equivalently in both years. The control lines are estimated for an adjusted sample size of 500.

None of the items had DIF which shows that the items function equivalently for patients independent of their gender, age, severity of diagnosis, number of consultations and survey year. The scatterplot in Figure 3 shows the item locations in the two surveys in 2004 and 2007. For the construction of this figure the two datasets were analysed separately. All item locations are within the upper and lower bonds of the 95% confidence interval as represented by the dotted lines.

**Figure 4:**
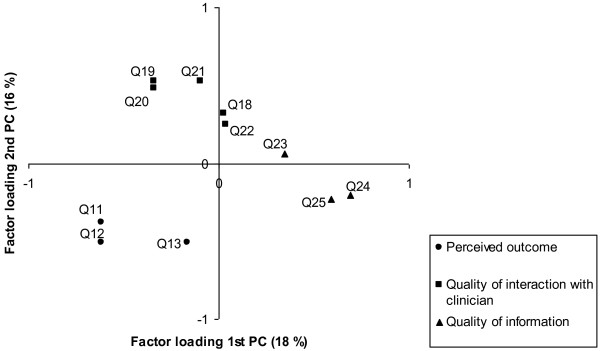
**Scatterplot of PCA**. Scatterplot of factor loadings for the two first principal components of the person-by-item residuals.

Although the major component of variance is well captured by the Rasch dimension, the principal component analysis of the person-by-item residuals indicated that there are two possibly very meaningful factors reflecting the original three aspects of care included in the POPEQ instrument - perceived outcome, quality of interaction with clinician and quality of information. Figure 4 is a scatterplot of the factor loading of these two first principal components for the initial analysis of all 11 items. As indicated by the labels of the axes in the figure, the components account for 18% and 16% of the variance in the residuals, respectively. Two subscales reflecting the loadings on the first residual component (items with positive and negative loadings, respectively) were formed and compared by independent t-tests [42]. 8.7% of the respondents had significantly different scores on the two subscales. The same procedure was followed for the second residual component resulting in 7.6% of the respondents with significantly different scores.

In Figure 4 different markers are used to identify which of the three aspects of care the item reflects. This illustrates how the slight tendency for multidimensionality of the data reflects the three different aspects of care originally used to develop the instrument. The first factor separates the three items relating to *quality of information *from the remaining items, while the second component separates the items relating to *quality of the interaction with the clinician*. The three subscales reflecting the three different aspects of care were computed, and subsequently the differences in score for the three subdimensions were tested by independent t-tests for all persons [42]. In the pairwise comparisons of differences between the three subdimensions the proportions of persons with significantly different subscores ranged from 6.9% to 9.0%.

## Discussion

The purpose of this article was to further assess the validity of the POPEQ, an 11-item measure of psychiatric out-patients experiences comprising three aspects of health care - the quality of interaction with the clinician, the perceived outcome of the treatment and the quality of information provision [19]. The POPEQ is part of a larger questionnaire used in Norwegian national surveys among psychiatric outpatients that provides information for national monitoring and benchmarking of specialized clinics offering psychiatric out-patient services.

Analyses based on classical test theory for the 2007 data confirmed previous results that the 11 item POPEQ instrument can be used to derive a valid and reliable measure of overall satisfaction with the quality of care in psychiatric out-patients clinics.

The Rasch analysis suggested that for two items the two lowest thresholds were so close to each other that they did not discriminate between patients, and hence, these thresholds were collapsed in the final calibration of the scale. Further, the outlier sensitive fit index, the outfit mean square, indicated that one item was slightly underfitting. However, the infit mean square fit index which weights respondents who are targeted by the item more strongly, suggested that the item works properly in separating patients with different levels of appraisal of their health care. Further, the effect of deleting or keeping this item is marginal. It is therefore recommended that the item should remain within the POPEQ to ensure content validity and maximize reliability.

The DIF analysis found that items were invariant for patients with different characteristics including gender, age, diagnosis groups, number of consultations and survey year. This is reassuring since the POPEQ is used to report a broad indicator of the quality of health care for specialized psychiatric out-patient clinics. In general these clinics have mixed composition of patients with these characteristics. The published scores for the institutions are case-mix adjusted to correct for such differences in composition. The lack of DIF along the most important variables in the case-mix model supports that the same construct is measured across subgroups with different characteristics. This provides further evidence that the case-mix model used is appropriate. The results of testing for DIF also support the temporal stability of the psychometric properties of the POPEQ items. The latter is particularly important since the measure is used as an indicator of change in quality of care for the psychiatric out-patient clinics. If the relation between the item and the construct changes over time and hence item drift is present, comparisons over time are less valid since the construct itself is not stable over time.

PCA of the residuals suggested that the 11 item scale to some degree reflects three subdimensions. These three subdimensions correspond to theoretically defined domains that the measure was intended to assess. However, the effect is quite small with 7-9% of the respondents having significantly different scores on the subscales. Our interpretation of this is that it is still meaningful to report the overall broad measure, but that it is also possible to develop three empirically separable and theoretically meaningful subscales reflecting *perceived outcome of the treatment *(3 items), *quality of interaction with the clinician *(5 items), and *quality of information provision *(3 items). These three subscales have person separation indexes around 0.8, which is reasonably high for scales with so few items. Items that are already included in the questionnaire may be assessed for their contribution to subscales. There are for instance four more items in the broader questionnaire relating to the perceived outcome for different types of treatment. These items were not possible to include in the original scale development based on classical test theory due to the fact that the items reflect types of treatment that many respondents have not received (e.g. treatment with medicines). Thus, the respondents were given the opportunity to respond 'not applicable' for these items. This created large proportions of systematic missing responses. However, one of the great advantages of Rasch analysis, is that respondents do not have to respond to exactly the same set of items. Similarly, the third subdimension, *quality of information provision*, may be developed with the inclusion of two more items relating to information about formal rights as a patient to complain and to gain access to the journal. The further development of these three subdimensions would lead to a greater breadth of measurement. Potentially, this may increase the perceived relevance of the quality indicators by providing the clinics with more targeted information facilitating local quality improvement efforts.

Results from national surveys including the POPEQ are used to develop quality indicators presented both to the public and to the responsible psychiatric institutions. Public use includes the internet site for free hospital choice in Norway. Research has shown that consumers have difficulties in understanding quality information [43], and that "less is more" in this respect [44]. Therefore, an aggregated and overall measure of satisfaction with the psychiatric out-patients clinics seems appropriate in the context of presenting information to consumers. On the other hand, more specific results are called for when reporting information to health providers aiming to evaluate and improve the quality of care [45]. Consequently, scores relating to the three POPEQ sub-dimensions might be a fruitful supplement when reporting results to the responsible psychiatric outpatient clinics.

## Conclusions

The application of Rasch analysis to the POPEQ has provided further evidence for the reliability and validity of the questionnaire as a measure of patient experiences of outpatient psychiatric care. The analysis showed that the 11 item scale is reasonably unidimensional, and it functions invariant across patients with different characteristics. Even if two items were rescored, a high level of internal consistency reliability was maintained. The POPEQ is recommended in future research relating psychiatric outpatients' experiences of care. The further development of three subscales reflecting different aspects of health care will be explored in future work. Beyond giving specific evidence for the validity of the POPEQ instrument, this analysis illustrates the added value of using Rasch analysis to inspect differential item functioning and to assess the dimensionality of instruments. Rasch models or other item response theory models are not frequently used in reporting scale development in patient satisfaction surveys, and hopefully this paper illustrates that this approach to scaling has several beneficial properties.

## Competing interests

The authors declare that they have no competing interests.

## Authors' contributions

RVO has conducted the analysis and he has also drafted the manuscript. AMG, HII and OAB have been involved in the development and design of the questionnaire used to collect the data. HII and OAB have been involved in the data acquisition. All authors have made significant contributions by critically reviewing the paper. All authors accept that the submitted version of the paper can be published.

## Pre-publication history

The pre-publication history for this paper can be accessed here:

http://www.biomedcentral.com/1472-6963/10/282/prepub

## Supplementary Material

Additional file 1 **The Psychiatric Out-Patient Experiences Questionnaire**. This document contains the complete questionnaire with the 11 items and response scales translated to English. This version does not reflect the original layout of the questionnaire. Click here for file
