# Supplementary material for: Rasch analysis of the Psychiatric Out-Patient Experiences Questionnaire (POPEQ)
Source: BMC Health Serv Res. 2010 Sep 28;10:282. doi: 10.1186/1472-6963-10-282 (PMC2955020; doi:10.1186/1472-6963-10-282)
Supplement: Additional file 1 — The Psychiatric Out-Patient Experiences Questionnaire. This document contains the complete questionnaire with the 11 items and response scales translated to English. This version does not reflect the original layout of the questionnaire. [file 1472-6963-10-282-S1.DOC]

# Appendix 1: Items from the questionnaire used in the POPEQ scale

|  | (Please cross one box for each question) | | No benefit | Small benefit | | Moderate benefit | | Large benefit | | Very large benefit | |  | |
| --- | --- | --- | --- | --- | --- | --- | --- | --- | --- | --- | --- | --- | --- |
| 11. | Have you benefited from consulting with a therapist at the out-patient clinic? | | □ | □ | | □ | | □ | | □ | |  | |
|  |  | |  |  | |  | |  | |  | |  | |
|  |  | | No benefit | Small benefit | | Moderate benefit | | Large benefit | | Very large benefit | |  | |
| 12. | What was the overall benefit of your therapy at the out-patient clinic? | | □ | □ | | □ | | □ | | □ | |  | |
|  |  | |  |  | |  | |  | |  | |  | |
|  |  | | Much  better | A little better | | Neither better nor worse | | A little worse | | Much worse | |  | |
| 13. | Compared to before your therapy started at the out-patient clinic, how is your psychological condition now? | | □ | □ | | □ | | □ | | □ | |  | |
|  | |  |  |  | |  | |  | |  | |  | |
|  | | To what extent... | Not at all | To a limited extent | | To a moderate extent | | To a large extent | | To a very large extent | |  | |
| 18. | | Was adequate time available for contacting and consulting with your therapist? | □ | □ | | □ | | □ | | □ | |  | |
| 19. | | Did your therapist understand your situation? | □ | □ | | □ | | □ | | □ | |  | |
| 20. | | Did your therapist take account of your situation? | □ | □ | | □ | | □ | | □ | |  | |
| 21. | | Did the therapist follow-up your therapy as planned? | □ | □ | | □ | | □ | | □ | |  | |
| 22. | | Were you able to tell your therapist about what you view as important about your situation? | □ | □ | | □ | | □ | | □ | |  | |
| 23. | | Were you involved in choosing the type of therapy? | □ | □ | | □ | | □ | | □ | |  | |
|  | |  |  | |  | |  | |  | |  | |  |
|  | |  | Very poor | | Fairly poor | | Neither poor nor good | | Fairly good | | Very good | | No opinion |
| 24. | | What did you think about the information you received about the therapy options available to you? | □ | | □ | | □ | | □ | | □ | | □ |
| 25. | | What do you think about the information you received about your psychological condition/diagnosis? | □ | | □ | | □ | | □ | | □ | | □ |
